# Supplementary figures and images for: Epigenetic aging differentially impacts breast cancer risk by self-reported race
Source: PLoS One. 2024 Oct 24;19(10):e0308174. doi: 10.1371/journal.pone.0308174 (PMC11500918; doi:10.1371/journal.pone.0308174)

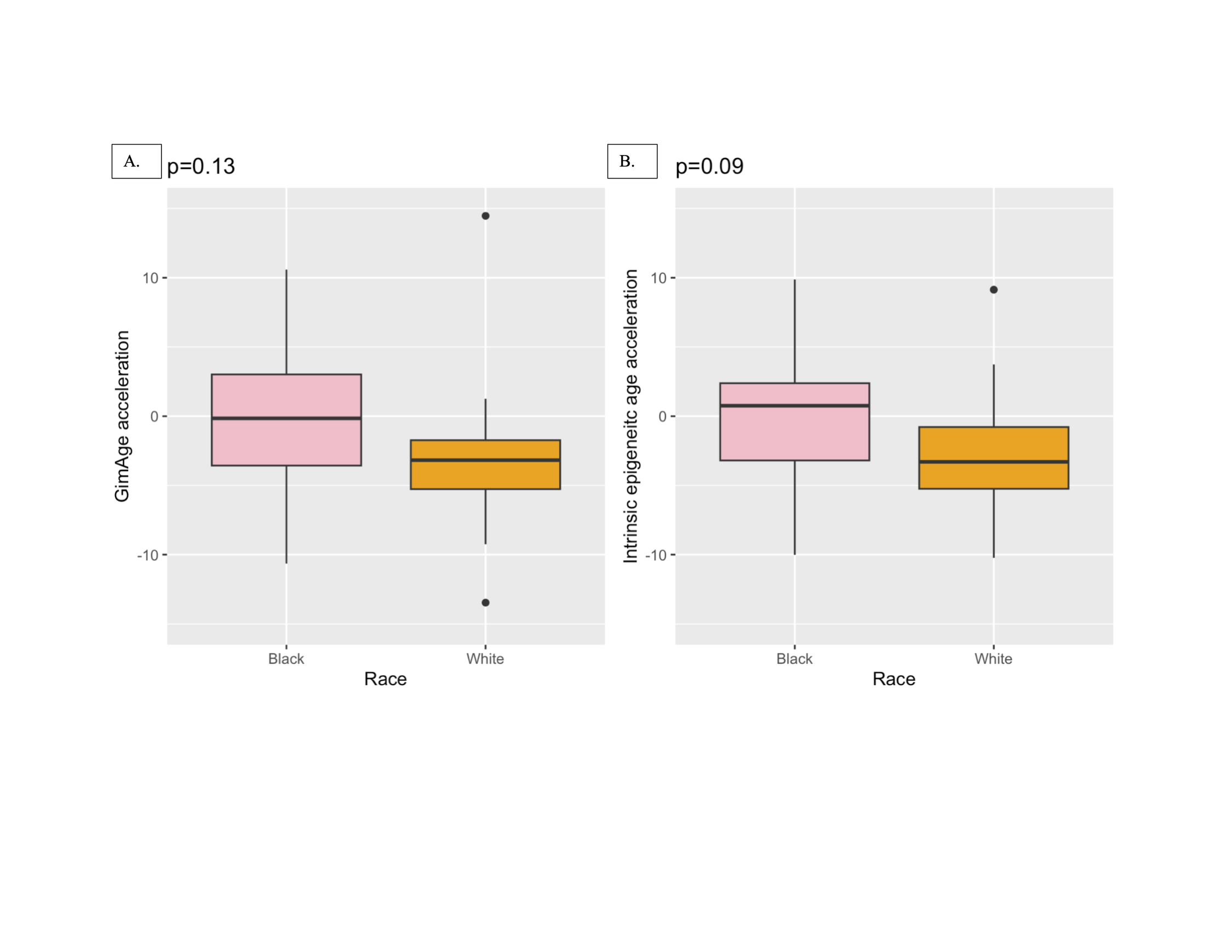

Supplement: S1 Fig — A. Boxplot of GrimAge acceleration in disease-free controls between Black and White, B. Boxplot of intrinsic epigenetic age acceleration in disease-free controls between Black and White. (TIF) [file pone.0308174.s001.tif]

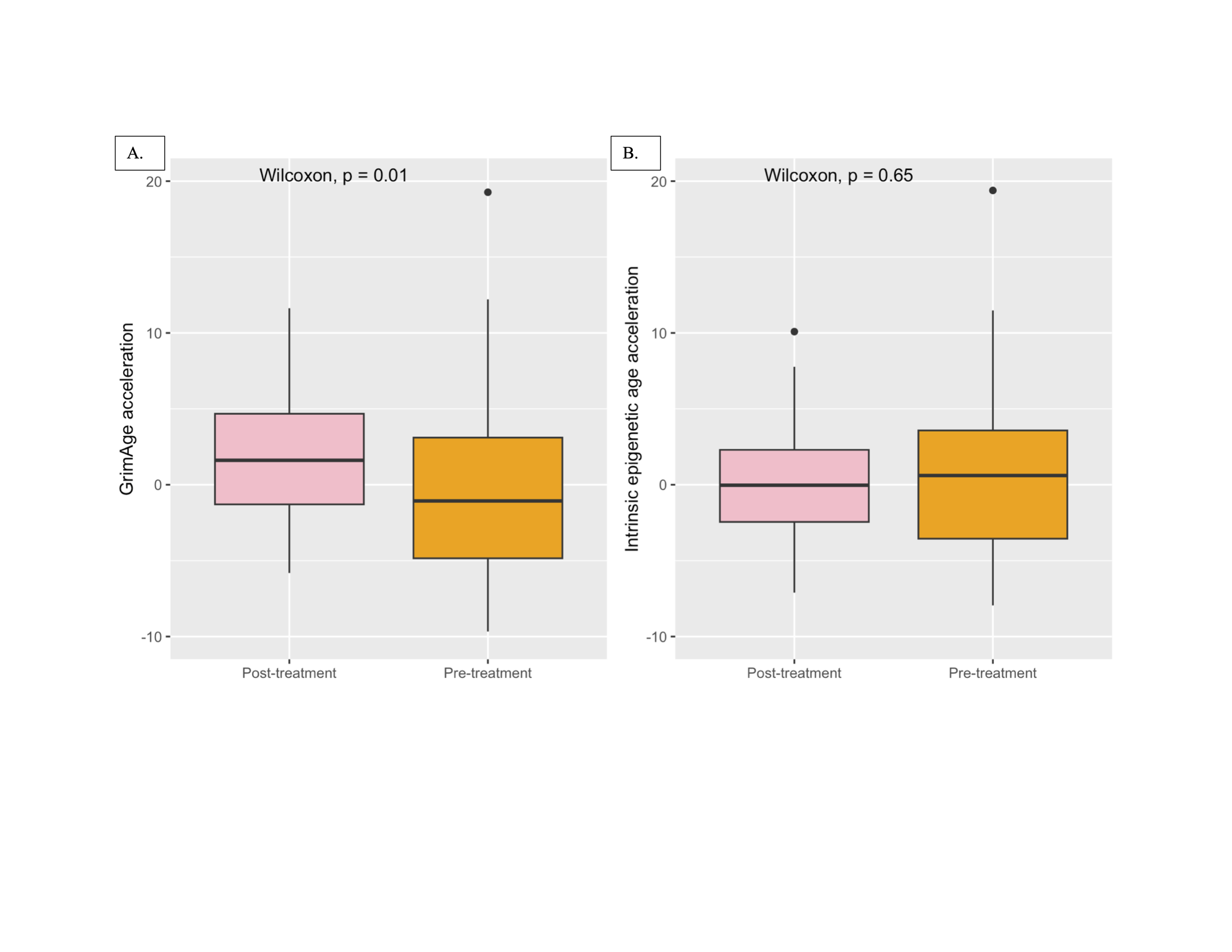

Supplement: S2 Fig — A. Boxplot of GrimAge acceleration between the DNA collections pre-treatment and post-treatment, B. Boxplot of intrinsic epigenetic age acceleration between the DNA collections pre-treatment and post-treatment. (TIF) [file pone.0308174.s002.tif]
