# Supplementary material for: Epigenetic aging differentially impacts breast cancer risk by self-reported race
Source: PLoS One. 2024 Oct 24;19(10):e0308174. doi: 10.1371/journal.pone.0308174 (PMC11500918; doi:10.1371/journal.pone.0308174)
Supplement: S1 Table — (DOCX) [file pone.0308174.s003.docx]

**S1 Table. The effect between different types of treatments on various epigenetic age acceleration measures**

|  |  | **OR(GrimAA)*** | **Pvalue** | **OR(IEAA)*** | **Pvalue** |
| --- | --- | --- | --- | --- | --- |
| Post-treatment | **Surgery only cases** (N=8)  vs controls (N=49) | 1.23 | 0.03 | 1.13 | 0.03 |
|  | **Chemotherapy only cases** (N=19)  vs controls (N=49) | 1.17 | 0.03 | 1.11 | 0.18 |
|  | **Radiotherapy only cases** (N=16)  vs controls (N=49) | 1.12 | 0.07 | 1.09 | 0.27 |
|  | **Chemotherapy and radiotherapy cases** (N=37)  vs controls (N=49) | 1.23 | 4x10^-4^ | 1.13 | 0.03 |
| Pre-treatment | **Pre-treatment cases** (N=47)  vs controls (N=49) | 1.05 | 0.20 | 1.11 | 0.03 |

*GrimAA* GrimAge acceleration; *IEAA* intrinsic epigenetic age acceleration; *OR* odds ratio

Note: * multivariable logistic regression model adjusting for age and self-reported race.
