# Supplementary material for: Epigenetic aging differentially impacts breast cancer risk by self-reported race
Source: PLoS One. 2024 Oct 24;19(10):e0308174. doi: 10.1371/journal.pone.0308174 (PMC11500918; doi:10.1371/journal.pone.0308174)
Supplement: S2 Table — (DOCX) [file pone.0308174.s004.docx]

**S2 Table. The associations between multiple epigenetic clocks and breast cancer risk**

|  | **OR^*^ [95% CI]** | **Pvalue** |
| --- | --- | --- |
| **GrimAA** | 1.14 [1.05, 1.25] | 2.86x10^-3^ |
| **DNAmAA** | 0.99 [0.95, 1.04] | 0.857 |
| **PhenoAA** | 1.01 [0.96, 1.04] | 0.963 |
| **DunedinPACE** | 1.37 [1.08, 1.79] | 0.014 |

*GrimAA* GrimAge acceleration; *DNAmAA* DNA methylation age acceleration (derived from Horvath clock); *PhenoAA* PhenoAge acceleration; *DunedinPACE* DNAm-based metrics of aging rates; *OR* odds ratio; *CI* confidence interval

Note: * multivariable logistic regression model adjusting for age, self-reported race and other known BrCa risk factors (BMI, age at menarche, parity, smoking status) as covariates.
