# Supplementary material for: Epigenetic aging differentially impacts breast cancer risk by self-reported race
Source: PLoS One. 2024 Oct 24;19(10):e0308174. doi: 10.1371/journal.pone.0308174 (PMC11500918; doi:10.1371/journal.pone.0308174)
Supplement: Attachment — Submitted filename: Response to Reviewer_final.docx [file pone.0308174.s005.docx]

**Response to Reviewer Critiques**

We express our gratitude to the editors and reviewers for their meticulous review and insightful comments on our manuscript, which have enhanced the quality of our work. Below, we address each reviewer's comments, followed by our responses marked in blue text.

**Reviewer #1**

Major concerns

1. There seems to be some confusion about the role of self-reported race in the analysis of GrimAge and breast cancer. The results from the stratified models and the significant interaction term suggest that race is a modifier of the association, not a confounder or a mediator as suggested by the authors.

Similar comments from Reviewer 2:

Line 158: I am not sure whether self-reported race would “confound” the association. It certainly is as effect measure modifier (EMM) but whether if fulfils the criteria for a confounder should be checked.

Response: We defined race as an effect modifier, given the results from the stratified analysis presented in Table 3 and our examination of the interaction effect between race and intrinsic epigenetic acceleration, and clarified this throughout the manuscript. We have made changes in lines 156-158. Furthermore, we have incorporated additional explanation regarding this aspect in lines 166-171 to provide more clarification.

2. It is perhaps not surprising that the association of intrinsic GrimAge and breast cancer is attenuated compared to the unadjusted model; Kresovich et al (2020, JAMA Network open) previously reported that blood cell composition is a marker of breast cancer risk suggesting that biological age and blood cell composition appear to detect overlapping, although not entirely, signals of breast cancer risk. This point should be made clear to readers.

Response: Thanks for highlighting this point. We have added the statement to our discussion section, as indicated on line 202-205.

3. The authors should report breast cancer associations with the DNAm proteins that comprise the GrimAge epigenetic clock. This would provide more of a basis to the comments made in the discussion where the authors suggest their associations are due to the underlying associations with cystatin C and leptin. (see Kresovich et al, Aging 2019)

Response: We have included the p-values for the association between cystatin C and leptin with breast cancer risk on line 193-195.

4. Although using the MethylationEPIC BeadChip information for biological age is interesting, there is a bit of a missed opportunity to perform an epigenome-wide association study of breast cancer at the individual CpG site resolution. A challenge would be replicating associations, but many EWASs of breast cancer have been performed, so the investigators could present an external validation of known CpG and BC associations.

Response: We agree and this is part of our future plans but beyond the scope of the currently presented project.

5. Similarly, the associations between other epigenetic clocks (Horvath, PhenoAge) and DNAm-based metrics of aging rates (DunedinPACE) should be at a minimum reported in supplemental material. There do not appear to be any papers between aging rates (DunedinPACE) and breast cancer, which would greatly strengthen the impact of this manuscript.

Similar comments from Reviewer 2:

Third, although it is understandable that the authors focus on GrimAge in this manuscript, it still would be interesting to see how the other epigenetic clocks (e.g. Horvath, Hannum, PhenoAge, DundinPACE) are associated with breast cancer. Additionally, this would increase comparability to other studies and substantially improve the impact of the study. I therefore suggest the authors provide these additional analyses as Supplementary Material and briefly discuss potential differences in the results. Since the authors used Steve Horvath’s website the other clocks should already be available.

Response: In response to these suggestions, we have incorporated DunedinPACE in our analysis. The associations between the Horvath clock, PhenoAge clock, and DNAm-based metrics of aging rates (DunedinPACE) with breast cancer risk have been reported in Supplemental Table S1 using model 3 (adjusting for known breast cancer risk factors). We have discussed these results in the discussion section line 207-217.

Minor concerns

6. There is some incorrect information about GrimAge in the discussion: the GrimAge clock is based on 7 DNAm-predicted plasma protein concentrations (not 88) and a DNAm predictor of smoking status, combined with chronological age and sex. The authors may need to revisit the original clock manuscripts to make sure their descriptions of the development procedures are correct.

Response: After reviewing the original GrimAge clock development paper, the typo in line 188-189 has been corrected.

7. The intrinsic GrimAge metric does not consider “white blood cell count” (line 200); instead, it accounts for “white blood cell composition” which is represented by the circulating proportion of different leukocyte subsets. Unfortunately, it is not possible to obtain reliable cell count information from methylation arrays.

Response: Thank you for bringing this to our attention. The calculation of the intrinsic age acceleration has been explained clearly in line 199-201.

8. The description of the figures seems to be incorrect. Please revise the figure images so the cases are pink and the disease-free controls are orange (opposite to what is noted in the figure legend).

Response: The legend of Figure 1 has been updated accordingly. Additionally, Figure 1 has been transformed into a box plot to convey more information effectively.

9. Please do not confuse self-reported race with ancestry. Ancestry is commonly measured using genetic data whereas race is often based on identity. Unless genetic data were used to inform the ancestry of the participants, please refer to the data as how the participants “self-identified” themselves.

Response: The confusions in the title and throughout the manuscript have been corrected.

**Reviewer #2**

Major concerns

10. First, I found the nomenclature sometimes confusing. I would suggest using the convention in the field as this would make it much easier to comprehend the information presented. Namely, the abbreviation AA_GrimAge and AA_GrimAge_In seem weird to me. In most studies you would find the following abbreviations:

- DNA methylation age: DNAmA

- DNA methylation age acceleration: DNAmAA

- GrimAge acceleration: GrimAA

- Intrinsic epigenetic age acceleration: IEAA

- Extrinsic epigenetic age acceleration: EEAA

Also, it is necessary to distinct better between methylation age and methylation age acceleration. Both are used interchangeably in the manuscript which is not correct (methylation age is the “raw” biological age measure and methylation age acceleration refers to the “gap” between chronological and biological age).

Response: Thank you for the feedback. We have updated the abbreviations in the manuscript for clarity.

Second, in some parts of the manuscripts the authors use causal language although causality is hard to deduct from this kind of (cross-section and observational) data. To confidently assume a causal effect, much more analyses and theoretical considerations need to be included (directed acyclic graph and others). Therefore, I would suggest rewriting the respective passages, e.g. exchange “results in” with “is associated with”.

Response: The wording has been changed throughout the manuscript.

Minor concerns:

Line 39: I think the convention is to use “intrinsic epigenetic age acceleration” when referring to the cell-type adjusted residuals. “GrimAge intrinsic clocks” sounds unfamiliar to me.

Line 44/45 “GrimAge intrinsic accelearion”: The same applies here. I think it would be better to stick to “intrinsic epigenetic age acceleration”.

Line 101: “intrinsic epigenetic age”: I think it should state “intrinsic epigenetic age acceleration”.

Line 103 “intrinsic epigenetic age” -> “intrinsic epigenetic age acceleration”

Line 106: “intrinsic age” -> “intrinsic age acceleration”

Line 137: Since you calculated a regression please exchange “correlated” with “associated”.

Line 138-140: I find this sentence confusing. The measure AA_Grim_In is per definition an cell-type adjusted residual of a regression of epigenetic age on chronological age. It therefore would not make sense to adjust any regression including AA_Grim_in for blood cell composition. I therefore would suggest that this sentence is rephrased, e.g. “similar effect size was found for the leukocyte cell type adjusted AA_Grim_In as well”.

Line 142: The word “impact” implies causation as well as a direction of the causal association. Neither can be deducted from cross-sectional data only. Please rephrase. Also this sentence is somewhat redundant to the second sentence in this paragraph.

Line 145: “effect” implies causation. Additionally, a potential effect of one variable on the other stays always the same. Only the effect size estimated from a statistical analysis changes.

Line 148 and 149: Please write “percentage points” instead of “%” if you are referring to the numerical difference between the risk.

Line 294: What do you mean by “imputed”? Were they measured and the missing values were imputed? Or were they estimated from the methylation data? Please clarify.

Line 274: “Data was processed for downstream analyses”: Please elaborate which quality control measures were used and how data was processed (software, packages).

Statistical Analysis: Please report which statistical software was used to conduct the analyses.

Figure 1 and S1: Please use boxplots instead of dynamite plots as a lot of information is lost. If possible, please show individual datapoints to illustrate the distribution.

List of abbreviations: IEAA and EEAA are noted but never used in the manuscript (although I would suggest to use these abbreviations instead of AA_GrimAge and AA_GrimAge_In).

Response: We have addressed all minor concerns regarding language and figure suggestions. We thank the reviewer for their suggestions.

Discussion: It is a frequently observed phenomenon that women have lower epigenetic age than chronological age (while men often have a higher epigenetic age). This is described frequently in the literature and might be important to mention in the discussion as this might not be known to all readers. Without context it could be confusing that the disease-free women are all epigenetically younger.

Response: We thank the reviewer for this comment and insight. Our analysis is restricted to women only; therefore the phenomenon of lower epigenetic aging would be present in both cases and controls to minimize bias.

Table 1: Please include the epigenetic age estimations in your table one (AA_GrimAge and AA_GrimAge_In).

Response: The GrimAge acceleration and intrinsic age acceleration estimates have been added to Table 1.

Table 3: Which of the models in Table 2 was used here? I think it would be interesting to show all three models of Table 2 also stratified by self-reported race.

Response: In Table 2 the results from model 3 were reported. A note has been added to Table 3 to illustrate this. In order to present succinct results we only present Model 3.

**Editor**

Journal Requirements:

When submitting your revision, we need you to address these additional requirements.

1. Please ensure that your manuscript meets PLOS ONE's style requirements, including those for file naming. The PLOS ONE style templates can be found at 
<https://journals.plos.org/plosone/s/file?id=wjVg/PLOSOne_formatting_sample_main_body.pdf> and 
<https://journals.plos.org/plosone/s/file?id=ba62/PLOSOne_formatting_sample_title_authors_affiliations.pdf>

2. Please update your submission to use the PLOS LaTeX template. The template and more information on our requirements for LaTeX submissions can be found at [http://journals.plos.org/plosone/s/latex](http://journals.plos.org/plosone/s/latex).

3. We note that the grant information you provided in the ‘Funding Information’ and ‘Financial Disclosure’ sections do not match.

When you resubmit, please ensure that you provide the correct grant numbers for the awards you received for your study in the ‘Funding Information’ section.

Grant numbers are now consistent.

4. Thank you for stating the following financial disclosure: 
"This study was funded by the National Cancer Institute (R03CA241956; P30CA043703)."

Please state what role the funders took in the study.  If the funders had no role, please state: ""The funders had no role in study design, data collection and analysis, decision to publish, or preparation of the manuscript."" 
If this statement is not correct you must amend it as needed. 
Please include this amended Role of Funder statement in your cover letter; we will change the online submission form on your behalf.

“The funders had no role in study design, data collection and analysis, decision to publish, or preparation of the manuscript.”

5. In this instance it seems there may be acceptable restrictions in place that prevent the public sharing of your minimal data. However, in line with our goal of ensuring long-term data availability to all interested researchers, PLOS’ Data Policy states that authors cannot be the sole named individuals responsible for ensuring data access (<http://journals.plos.org/plosone/s/data-availability#loc-acceptable-data-sharing-methods>).

Data requests to a non-author institutional point of contact, such as a data access or ethics committee, helps guarantee long term stability and availability of data. Providing interested researchers with a durable point of contact ensures data will be accessible even if an author changes email addresses, institutions, or becomes unavailable to answer requests.

Before we proceed with your manuscript, please also provide non-author contact information (phone/email/hyperlink) for a data access committee, ethics committee, or other institutional body to which data requests may be sent. If no institutional body is available to respond to requests for your minimal data, please consider if there any institutional representatives who did not collaborate in the study, and are not listed as authors on the manuscript, who would be able to hold the data and respond to external requests for data access? If so, please provide their contact information (i.e., email address). Please also provide details on how you will ensure persistent or long-term data storage and availability.

Currently, our institutions lack the infrastructure for this request.

6. We notice that your supplementary figure S1 is included in the manuscript file. Please remove them and upload them with the file type 'Supporting Information'. Please ensure that each Supporting Information file has a legend listed in the manuscript after the references list.

We have moved Supplementary Figure S1 to the supporting information file.
