# Supplementary material for: Epigenetic aging differentially impacts breast cancer risk by self-reported race
Source: PLoS One. 2024 Oct 24;19(10):e0308174. doi: 10.1371/journal.pone.0308174 (PMC11500918; doi:10.1371/journal.pone.0308174)
Supplement: Attachment — Submitted filename: Response to Reviewers_frs.docx [file pone.0308174.s006.docx]

**Response to Reviewers**

*We thank the reviewers and editors for their time and consideration of our manuscript. Please find our response to the critiques and comments provided.*

Reviewer #3: There is one issue with the analysis that has not been addressed. The differences in age acceleration being greater in the breast cancer patients appears real but the authors must take into account the effect of chemotherapy on age acceleration as this would skew any results in favor of greater age acceleration which is not due to breast cancer per se. So, in the analysis this must be controlled for.s

*Response: Thank you for this comment. We have included several additional analyses addressing this important concern and strengthening our manuscript.*

*In the revised manuscript, breast cancer treatment status at DNA collection was added to the text and Table 1 (excerpt; data added). Among breast cancer cases, pre-treated DNA was collected from 47 (31.5%) women and post-treated DNA from 102 (68.5%) women. The distribution of treatment types for DNA collected post-treatment is presented in Table 1. Treatment type was known for ~80% of DNA collected post-treatment. A DNA sample was collected following chemotherapy or a combination of chemotherapy and radiotherapy in approximately 55% of the women. Post-treated DNA samples were collected in 8% of the women who underwent surgery only, whereas treatment status was missing for slightly over 20% of the women. The missing treatment data may impact our results. We have added this to the discussion.*

Table 1 (new data):

| Treatment status at DNA collection | Breast cancer cases | Disease-free controls |
| --- | --- | --- |
| Pre-treatment | 47 (31.5%) | NA |
| Post-treatment | 102 (68.5%) | NA |
| Surgery only | 8 (7.8%) | NA |
| Chemotherapy only | 19 (18.6%) | NA |
| Radiotherapy only | 16 (15.7%) | NA |
| Both chemotherapy and radiotherapy | 37 (36.3%) | NA |
| Missing | 22 (21.6%) | NA |

*To assess the impact of breast cancer treatment on the association of age acceleration (AA) measures, we compared the mean AA by treatment status at DNA collection using the Wilcoxon test. The results, shown in the following figure, indicate post-treatment GrimAge acceleration (GrimAA) was 1.79 units greater than pre-treated GrimAA (p = 0.003), however* ***no mean difference was observed between the post- and pre-treatment groups for intrinsic epigenetic age acceleration (IEAA) (p=0.80)****.*

*Based on these results, our data suggests that breast cancer treatment has less impact on IEAA. This result demonstrates the robustness of our conclusions and has been observed in the published literature^1-2^, thus suggesting that IEAA may be less sensitive to unaccounted factors, thereby mitigating the effects of residual confounding. We have added this statement to the discussion section, as indicated on line 439. And the above figure will be named as S2 Fig and added to the manuscript.*

*We also compared the effect of different types of treatments on GrimAA and IEAA among those patients whose samples were collected post-treatment. We used a different subset of breast cancer cases by different types of treatments and compared them to the same set of disease-free controls to assess effects (ORs) across treatment groups. Multivariable logistic regression models were conducted, adjusting for age and self-reported race (Model 2). The results were shown in the Table below and have also been added to the manuscript as S1 Table.*

|  |  | OR(GrimAA) | Pvalue | OR(IEAA) | Pvalue |
| --- | --- | --- | --- | --- | --- |
| Post-treatment | Surgery only cases (N=8)  vs controls (N=49) | 1.23 | 0.03 | 1.13 | 0.03 |
|  | Chemotherapy only cases (N=19)  vs controls (N=49) | 1.17 | 0.03 | 1.11 | 0.18 |
|  | Radiotherapy only cases (N=16)  vs controls (N=49) | 1.12 | 0.07 | 1.09 | 0.27 |
|  | Chemotherapy and radiotherapy cases (N=37)  vs controls (N=49) | 1.23 | 4x10^-4^ | 1.13 | 0.03 |
| Pre-treatment | Pre-treatment cases (N=47)  vs controls (N=49) | 1.05 | 0.20 | 1.11 | 0.03 |

*Overall, the IEAA OR is very consistent across all groups (range: 1.09-1.13), further suggesting that treatment had minimal impact on DNA collected following treatment. It is worth noting, the GrimAA ORs had more variation and ranged from 1.05-1.23. Most likely this is an artifact of our limited sample size.*

*Here, we limited our analysis to Model 2 adjusting for age and self-reported race based on our limited sample size. Furthermore, the effect estimates from Model 2 presented in Table 2 with pre- and post-treated DNA samples combined were nearly identical, indicating either very minimal or no confounding from including additional breast cancer risk factors.*

Citations

1. Brägelmann, Johannes, and Justo Lorenzo Bermejo. "A comparative analysis of cell-type adjustment methods for epigenome-wide association studies based on simulated and real data sets." Briefings in Bioinformatics 20.6 (2019): 2055-2065.
2. Pottinger, Tess D., et al. "Association of cardiovascular health and epigenetic age acceleration." Clinical epigenetics 13 (2021): 1-6.
